# Case Report: Dramatic response of trastuzumab rezetecan in an NSCLC patient with EGFR sensitive mutation and primary ERBB2 genetic alterations

**DOI:** 10.3389/fonc.2026.1941676

**Published:** 2026-09-16

**Authors:** Lin Zhang, Chuantao Zhang, Huiyun Wang, Qingxia Li, Yongjie Wang, Xiao Huang, Dantong Sun, Helei Hou, Lijian Zhang

**Affiliations:** 1 Department of Oncology, The Affiliated Hospital of Qingdao University, Qingdao, Shandong, China; 2 Department of Thoracic Surgery, The Affiliated Hospital of Qingdao University, Qingdao, Shandong, China; 3 Department of Medical Oncology, Peking University First Hospital, Peking University, Beijing, China

**Keywords:** brain metastasis, EGFR mutation, ERBB2 amplification, non-small cell lung cancer, trastuzumab rezetecan

## Abstract

Epidermal growth factor receptor tyrosine kinase inhibitors (EGFR-TKIs) have provided significant clinical benefit to patients with EGFR-mutant non-small cell lung cancer (NSCLC), but multiple concurrent genetic alterations may limit their efficacy. We report a case of advanced lung adenocarcinoma harboring concurrent EGFR exon 19 deletion, ERBB2 amplification, and ERBB2 p.S310F mutation. The patient experienced disease progression after multiple lines of therapy, including osimertinib-based treatment, pemetrexed/carboplatin plus ivonescimab, and nab-paclitaxel plus Endostar. Considering that primary ERBB2 genetic alterations might contribute to the limited benefit from EGFR-TKI-based therapy, the patient subsequently received trastuzumab rezetecan monotherapy. After four cycles of treatment, the patient achieved a partial response in pulmonary lesions and a complete response in intracranial metastases. This case indicates that trastuzumab rezetecan may be a promising treatment option for EGFR-mutant NSCLC with concurrent ERBB2 genetic alterations, even after failure of multiple lines of treatment.

## 1 Introduction

Lung cancer remains the leading cause of cancer-related death worldwide (1), and non-small cell lung cancer (NSCLC) accounts for approximately 85% of all cases (2). In NSCLC, epidermal growth factor receptor (EGFR) mutations represent one of the key oncogenic drivers, particularly in Asian patients with lung adenocarcinoma, where the mutation rate reaches 40%–60% (3, 4). The development of EGFR tyrosine kinase inhibitors (EGFR-TKIs), especially third-generation agents, has substantially improved progression-free survival, overall survival, and quality of life in patients with EGFR-mutant NSCLC (5).

Resistance to EGFR-targeted therapy is inevitable, and the mechanisms are highly heterogeneous and include secondary EGFR mutations, histologic transformation, and activation of bypass pathways involving genes such as MET, ERBB2, and AXL (6–8). Among these, ERBB2 mutations and amplification, together with HER2 protein overexpression, have increasingly been recognized as important mechanisms of resistance to EGFR-TKIs (9, 10). Notably, ERBB2 alterations may arise as acquired resistance events during treatment, but may also be present at baseline as co-existing genomic abnormalities that potentially limit the efficacy of EGFR-TKI therapy from the outset (11, 12). Trastuzumab rezetecan, a HER2-targeting antibody-drug conjugate (ADC), has demonstrated remarkable activity in ERBB2-mutant NSCLC (13). Here, we report a case of NSCLC harboring a primary EGFR-sensitive mutation alongside ERBB2 amplification and ERBB2 p.S310F mutation, which was resistant to osimertinib but achieved a marked response to trastuzumab rezetecan following failure of multiple lines of treatment.

## 2 Case presentation

A 58-year-old woman first presented in December 2023 with symptoms of dry cough for 1 month. A chest CT scan showed a left upper lobe lesion suspicious for primary lung cancer (**Figure 1A**). PET/CT showed a hypermetabolic soft-tissue mass in the left hilar region with metastatic involvement of multiple lymph node stations and the pleura. Histopathological examination of an ultrasound-guided biopsy of a left cervical lymph node showed metastatic adenocarcinoma. In the context of the dominant pulmonary mass and the radiographic pattern of spread, the patient was clinically diagnosed with stage IV lung adenocarcinoma. Brain MRI at initial diagnosis revealed a metastatic lesion in the right cerebellar hemisphere (**Figure 1A**). Targeted next-generation sequencing was performed on formalin-fixed, paraffin-embedded tumor tissue obtained from an ultrasound-guided biopsy of a metastatic left cervical lymph node at initial diagnosis in December 2023. A 196-gene targeted panel provided by Geneseeq Technology Inc. (Nanjing, China) was used. The mean sequencing depth was ≥500×, with an effective sequencing depth of ≥200×. The assay applied tiered variant allele frequency (VAF) thresholds according to variant type and hotspot category, as detailed in **Supplementary Tables S1**, **S2**. Copy-number amplification was defined as CN >3.6. The assay identified concurrent EGFR exon 19 deletion, ERBB2 p.S310F mutation, and ERBB2 amplification, among others. Detailed genomic findings are summarized in **Table 1**. Given the presence of an EGFR-sensitive mutation, the patient received first-line osimertinib 80 mg once daily in December 2023. The best radiographic response to osimertinib monotherapy was stable disease (SD), which was maintained for approximately 5 months. Despite radiographic disease stability, the serum carcinoembryonic antigen (CEA) level increased from 79.5 to 103.2 ng/mL (**Figure 2**), suggesting limited clinical benefit. The patient subsequently received pemetrexed/carboplatin (PC) plus osimertinib in May 2024. After four cycles of treatment, the patient achieved systemic disease control with a decline in CEA levels. Brain MRI in July 2024 also showed a decrease in the size of the right cerebellar metastasis compared with baseline (**Figure 1A**). Because of residual primary disease, the patient initiated concurrent thoracic intensity-modulated radiotherapy (IMRT) combined with etoposide–cisplatin (EP) chemotherapy in October 2024, while osimertinib was continued. Unfortunately, imaging performed after the completion of radiotherapy in January 2025 revealed progressive disease (PD), with an increase in both the number and size of scattered nodules in the bilateral lungs on chest CT and enlargement of the pre-existing metastatic lesion in the right cerebellar hemisphere on brain MRI (**Figure 1A**). Osimertinib was then discontinued, and the patient received second-line PC plus ivonescimab. After two cycles, brain MRI revealed further progression of intracranial metastases, and the response was assessed as PD (**Figure 1B**). Third-line nab-paclitaxel plus Endostar was subsequently administered. After five cycles, brain MRI confirmed marked intracranial progression. Brain IMRT with concurrent oral temozolomide was then given, resulting in regression of irradiated intracranial lesions. Nevertheless, systemic disease progressed again, with CEA rising from 21.46 to 43.07 ng/mL (**Figure 2**) and thoracic imaging showing enlargement and increase of pulmonary metastases (**Figure 1C**). Considering progression after multiple prior therapies and the presence of baseline ERBB2 alterations, the patient started fourth-line trastuzumab rezetecan monotherapy in September 2025. Imaging after brain radiotherapy and before initiation of fourth-line trastuzumab rezetecan is shown in **Figure 1D**. At treatment initiation, she weighed 65 kg. Based on the HORIZON-Lung regimen of 4.8 mg/kg every 3 weeks, the calculated dose was 312 mg; therefore, a rounded dose of 300 mg was administered intravenously every 3 weeks, corresponding to an actual dose of 4.62 mg/kg per cycle. Remarkably, after four cycles, restaging imaging revealed a profound and rapid response. Chest CT demonstrated near-complete resolution of pulmonary metastases and marked regression of the primary lesion, achieving a partial response (PR). Simultaneously, brain MRI showed complete resolution of all previously identified intracranial metastatic lesions, indicating an intracranial complete response (CR) (**Figure 1E**). This response was accompanied by a marked decline in serum CEA levels, which decreased from 43.07 to 10.9 ng/mL within the first month of treatment (**Figure 2**). During trastuzumab rezetecan treatment, the patient experienced anorexia, anemia, nausea, and vomiting. After the fourth cycle, the white blood cell count decreased to 0.98 × 10^9^/L, corresponding to grade 4 leukopenia according to CTCAE version 5.0; the leukopenia improved after supportive treatment. The treatment timeline for this patient is shown in **Figure 3**. Written informed consent was obtained from the patient for publication of this case and accompanying images.

**Figure 1:**
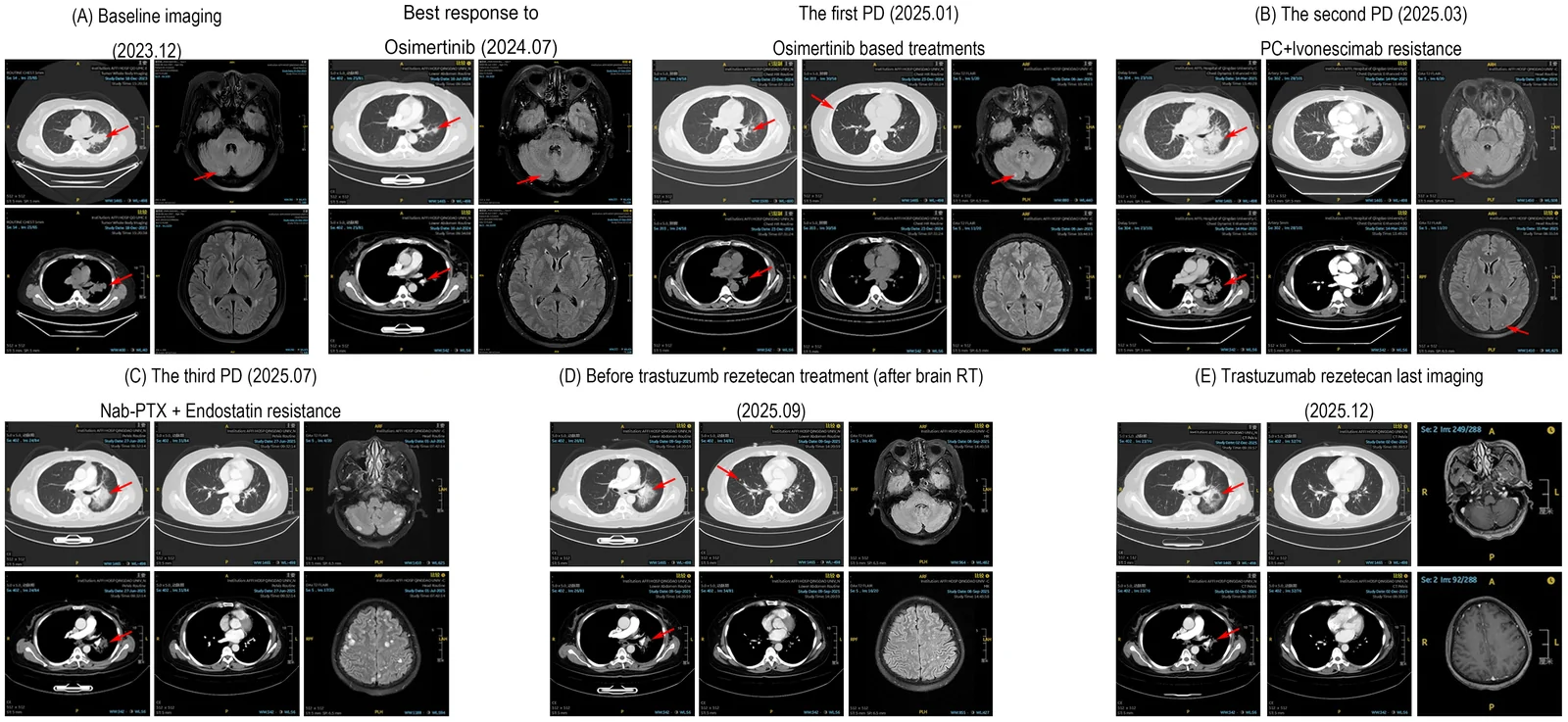
Serial chest CT and brain MRI findings throughout treatment. **(A)** Baseline imaging, the best response to first-line osimertinib-based therapy, and the first progression during osimertinib-based treatment. **(B)** Disease progression on second-line therapy with PC chemotherapy (pemetrexed combined with carboplatin) plus ivonescimab. **(C)** Disease progression on third-line nab-paclitaxel plus Endostar. **(D)** Images after brain radiotherapy and before initiation of fourth-line trastuzumab rezetecan. **(E)** Last imaging during trastuzumab rezetecan therapy.

**Table 1:** Baseline next-generation sequencing (NGS) findings at initial diagnosis.

Gene	Alteration	Reported value
EGFR	Exon 19 p.L747_P753delinsS (exon 19 deletion)	VAF 25.88%
ERBB2	Exon 8 p.Ser310Phe (p.S310F) missense mutation	VAF 87.32%
ERBB2	Gene copy number amplification	CN 10.3
TP53	Exon 4 p.Trp53Ter (p.W53*) nonsense mutation	VAF 44.04%
TOP2A	Copy number gain	CN 4.5

VAF, variant allele frequency; CN, copy number.

**Figure 2:**
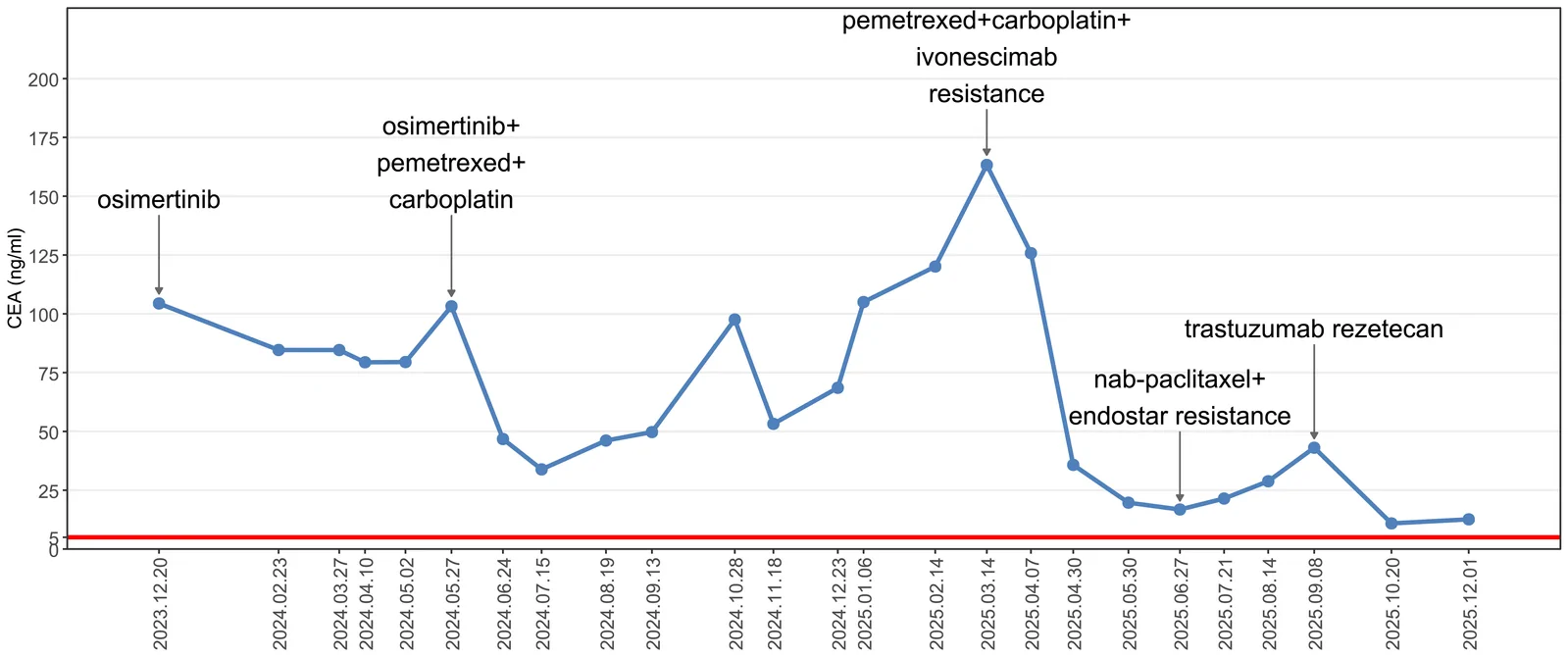
Dynamic changes in serum carcinoembryonic antigen (CEA) levels during the course of treatment. The red horizontal line indicates the upper limit of normal (5 ng/mL).

**Figure 3:**
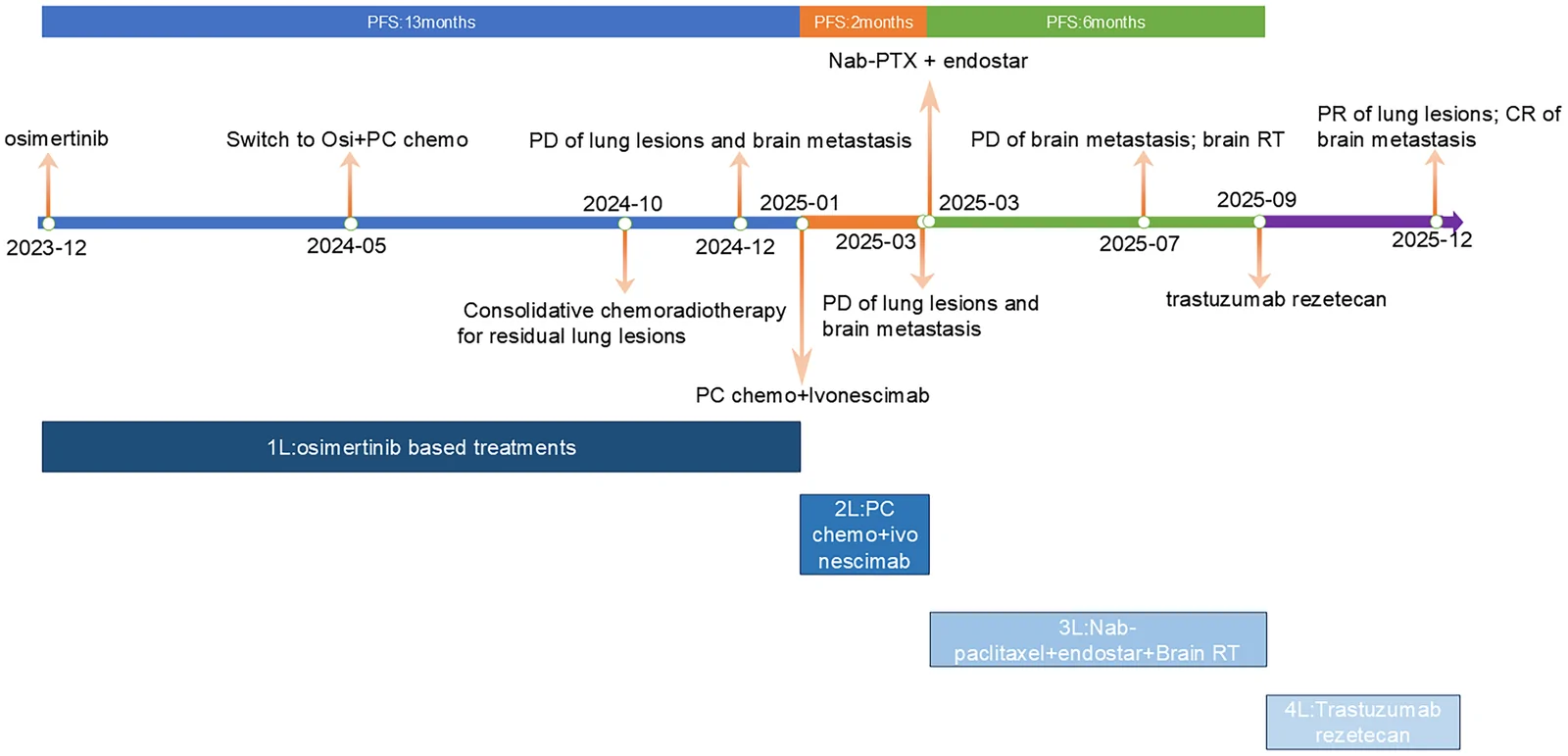
Timeline of patient treatment milestones. The interval from December 2023 to January 2025 represents the overall progression-free interval during sequential osimertinib-based treatment.

## 3 Discussion

This case highlights the potential clinical significance of concurrent ERBB2 alterations in EGFR-mutant NSCLC. ERBB2 alterations, including amplification and activating mutations, have been associated with poorer responses to EGFR-TKIs and inferior prognosis. In this case, osimertinib monotherapy achieved stable disease for approximately 5 months, after which chemotherapy and subsequently thoracic chemoradiotherapy were added. The total progression-free interval from the initiation of osimertinib to documented disease progression was 13 months, suggesting that the overall clinical benefit of this sequential osimertinib-based treatment strategy was suboptimal. ERBB2-driven bypass signaling might have contributed to the limited benefit from osimertinib-based therapy. In a cohort of 200 patients with EGFR-mutant NSCLC, Yu et al. reported that baseline ERBB2 amplification was independently associated with shorter progression-free survival after EGFR-TKI treatment (11). Thus, although only 1.5% of EGFR-mutant tumors harbor concurrent ERBB2 alterations, identifying this baseline molecular profile is clinically important given the large population of lung cancer patients (14).

Treatment options after EGFR-TKI resistance are no longer limited to conventional chemotherapy. ORIENT-31 and HARMONi-A showed improved progression-free survival with combination regimens including immunotherapy and antiangiogenic therapy in patients with EGFR-mutant NSCLC after EGFR-TKI failure (15, 16). However, our patient experienced rapid progression after chemotherapy plus ivonescimab, suggesting that patients with ERBB2-driven resistance to EGFR-TKIs may not derive benefit from this regimen. The TROP2-directed sacituzumab tirumotecan has shown promising efficacy in previously treated EGFR-mutant NSCLC (17), while trastuzumab deruxtecan has established clinical benefit in ERBB2-mutant NSCLC in the DESTINY-Lung trial (18, 19). Trastuzumab rezetecan is another HER2-directed ADC with promising activity in ERBB2-mutant NSCLC, as demonstrated in the HORIZON-Lung study (13). The subsequent response to trastuzumab rezetecan suggests that for patients with identifiable primary or acquired resistance mechanisms to EGFR-TKIs such as ERBB2 alterations, a mechanism-based therapeutic strategy may be superior to conventional or standard treatment.

In our report, fourth-line trastuzumab rezetecan monotherapy induced marked regression of the primary pulmonary lesion and pulmonary metastases, accompanied by a substantial decrease in serum CEA, providing the strongest evidence of systemic antitumor activity of the ADC. In contrast, interpretation of the intracranial response is more complex because the patient had received brain IMRT with concurrent oral temozolomide shortly before trastuzumab rezetecan, and radiographic responses after cranial irradiation may continue to evolve over subsequent weeks to months (20, 21). Although complete radiographic resolution of the intracranial lesions was observed during trastuzumab rezetecan treatment, this finding cannot be definitively attributed to the ADC alone and may have resulted from prior brain radiotherapy, trastuzumab rezetecan, or their combined effects.

ERBB2 p.S310F is an activating mutation located in the extracellular domain of HER2. Functional studies have demonstrated that this alteration promotes receptor activation and downstream signaling and can facilitate the formation of an active heterodimer with EGFR (22, 23). Although unconjugated trastuzumab may not adequately suppress S310F-mediated signaling, experimental studies indicate that its binding to the mutant receptor is retained (23). This feature is relevant to trastuzumab-based ADCs, whose antitumor activity depends not only on inhibition of HER2 signaling but also on antibody binding, receptor-mediated internalization, and intracellular delivery of a cytotoxic payload (24). Concurrent ERBB2 amplification may further increase gene dosage and potentially enhance the availability of HER2 receptors for ADC binding and internalization. In this case, ERBB2 amplification with a high copy number and the ERBB2 S310F mutation were simultaneously identified by NGS. However, neither HER2 immunohistochemistry nor ERBB2 fluorescence *in situ* hybridization was performed because of insufficient residual biopsy tissue for additional testing. In addition, the membrane-permeable topoisomerase I inhibitor payload of trastuzumab rezetecan may exert a bystander effect in adjacent tumor cells with heterogeneous HER2 expression (25). Further studies are needed to clarify this effect.

In DESTINY-Lung02, grade 3 or higher drug-related adverse events occurred in 39.6% and 60.0% of patients receiving trastuzumab deruxtecan at 5.4 and 6.4 mg/kg, respectively, whereas adjudicated drug-related ILD occurred in 14.9% and 32.0%, including one fatal event in each dose group (26). Trastuzumab rezetecan also has clinically relevant toxicities. In HORIZON-Lung, the most common grade 3–4 treatment-related adverse events were decreased neutrophil count, decreased white blood cell count, anemia, and decreased platelet count, occurring in 40%, 27%, 23%, and 11% of patients, respectively. A relatively low incidence of treatment-related serious ILD (5%) was reported, with no treatment-related deaths observed (13). Although trastuzumab deruxtecan would also have represented a reasonable therapeutic option, the patient’s prior thoracic radiotherapy raised concern regarding the potential overlap between radiation-related lung injury and ADC-associated pulmonary toxicity. Therefore, based on this individualized pulmonary risk assessment and the greater clinical accessibility of trastuzumab rezetecan in our setting, trastuzumab rezetecan was selected for this patient. During trastuzumab rezetecan treatment, the patient experienced anorexia, anemia, nausea, and vomiting, and developed grade 4 leukopenia after the fourth cycle, with a white blood cell count of 0.98 × 10^9^/L; the leukopenia improved after supportive treatment. This adverse-event profile is consistent with the predominantly hematologic toxicity observed in HORIZON-Lung (13). In this case, trastuzumab rezetecan monotherapy was selected instead of combination therapy with osimertinib because of concerns about overlapping toxicities, particularly interstitial lung disease or pneumonitis. The efficacy and safety of combining HER2-directed ADCs with EGFR-TKIs require further investigation.

With the increasing use of NGS, the coexistence of multiple actionable oncogenic alterations poses a real-world challenge in identifying the dominant driver and prioritizing first-line targeted therapy. In this case, the VAF of ERBB2 p.S310F (87.32%) was substantially higher than that of the EGFR exon 19 deletion (25.88%), raising the question of which alteration represented the dominant oncogenic driver. However, VAF alone cannot determine biological dominance, and its interpretation should also consider the oncogenic function, clonality of each alteration and the available clinical evidence (27). Despite the higher VAF of ERBB2 p.S310F, the EGFR exon 19 deletion is a well-established sensitizing alteration, supporting the propriety of using osimertinib as first-line treatment. In future clinical practice, treatment decisions in similar cases should integrate molecular features and clinical evidence, with close monitoring and timely adjustment to maximize clinical benefit.

## 4 Conclusion

This case suggests that trastuzumab rezetecan may represent a promising treatment option for EGFR-mutant NSCLC with primary ERBB2 alterations after EGFR-TKI failure. It also raises the possibility that HER2-directed ADC therapy may have clinical value in patients with acquired ERBB2 alterations following EGFR-TKI resistance. More importantly, when actionable EGFR and ERBB2 alterations coexist at baseline, the selection of first-line targeted therapy should consider VAF together with other molecular features and clinical evidence.

## Data Availability

The original contributions presented in the study are included in the article/**Supplementary Material**. Further inquiries can be directed to the corresponding authors.
